# Association of Nonsteroidal Anti-inflammatory Drug Prescriptions With Kidney Disease Among Active Young and Middle-aged Adults

**DOI:** 10.1001/jamanetworkopen.2018.7896

**Published:** 2019-02-15

**Authors:** D. Alan Nelson, Eric S. Marks, Patricia A. Deuster, Francis G. O’Connor, Lianne M. Kurina

**Affiliations:** 1 Division of Primary Care and Population Health, Department of Medicine, Stanford University School of Medicine, Stanford, California; 2 Division of Nephrology, Department of Medicine, Uniformed Services University, Bethesda, Maryland; 3 Consortium for Health and Military Performance, Department of Military and Emergency Medicine, Uniformed Services University, Bethesda, Maryland

## Abstract

**Importance:**

Concern about the renal effects of nonsteroidand al anti-inflammatory drugs (NSAIDs) among young, healthy adults has been limited, but more attention may be warranted given the prevalent use of these agents.

**Objective:**

To test for associations between dispensed NSAIDs and incident acute kidney injury and chronic kidney disease while controlling for other risk factors.

**Design, Setting, and Participants:**

This retrospective, longitudinal cohort study used deidentified medical and administrative data on 764 228 active-duty US Army soldiers serving between January 1, 2011, and December 31, 2014. Analysis was conducted from August 1 to November 30, 2018. All individuals new to Army service were included in the analysis. Persons already serving in January 2011 were required to have at least 7 months of observable time to eliminate those with kidney disease histories.

**Exposures:**

Mean total defined daily doses of prescribed NSAIDs dispensed per month in the prior 6 months.

**Main Outcomes and Measures:**

Incident outcomes were defined by diagnoses documented in health records and a military-specific digital system.

**Results:**

Among the 764 228 participants (655 392 [85.8%] men; mean [SD] age, 28.6 [7.9] years; median age, 27.0 years [interquartile range, 22.0-33.0 years]), 502 527 (65.8%) were not dispensed prescription NSAIDs in the prior 6 months, 137 108 (17.9%) were dispensed 1 to 7 mean total defined daily doses per month, and 124 594 (16.3%) received more than 7 defined daily doses per month. There were 2356 acute kidney injury outcomes (0.3% of participants) and 1634 chronic kidney disease outcomes (0.2%) observed. Compared with participants who received no medication, the highest exposure level was associated with significantly higher adjusted hazard ratios (aHRs) for acute kidney injury (aHR, 1.2; 95% CI, 1.1-1.4) and chronic kidney disease (aHR, 1.2; 95% CI, 1.0-1.3), with annual outcome excesses per 100 000 exposed individuals totaling 17.6 cases for acute kidney injury and 30.0 cases for chronic kidney disease.

**Conclusions and Relevance:**

Modest but statistically significant associations were noted between the highest observed doses of NSAID exposure and incident kidney problems among active young and middle-aged adults.

## Key Points

**Question:**

What is the association between prescribed dosages of nonsteroidal anti-inflammatory drugs and later incident kidney disease among active young and middle-aged adults?

**Findings:**

In this cohort study of 764 228 US Army soldiers, prescriptions of more than 7 daily defined doses of nonsteroidal anti-inflammatory drugs per month were associated with modest but significant increases in the adjusted hazard ratios of acute and chronic kidney disease diagnoses.

**Meaning:**

Prescribers should be cognizant of potential kidney disease risks associated with higher doses of nonsteroidal anti-inflammatory drugs among active young and middle-aged adults; dosage reduction represents an approach that may decrease associated kidney disease outcome rates.

## Teaser

This cohort study evaluates the association between use of nonsteroidal anti-inflammatory drugs and incident acute kidney injury and chronic kidney disease in US Army soldiers.

## Introduction

Nonsteroidal anti-inflammatory drugs (NSAIDs) are widely used in the United States in prescription and over-the-counter forms,^1^ with more than 70 million NSAID prescriptions written annually.^2^ In 2010, more than 29 million US adults were estimated to be regular NSAID users—an increase of 41% from 2005.^3^ A recent study of self-reported over-the-counter and prescribed ibuprofen therapy noted that 90% of those using ibuprofen took it regularly, 37% took another NSAID in addition to ibuprofen, and 11% exceeded the recommended daily limit of ibuprofen.^4^

Clinicians who prescribe or recommend NSAIDs should weigh the benefits vs the risks for kidney health. Both selective and nonselective NSAIDs adversely affect the kidneys through prostaglandin-related effects.^5^ Potential insults include impaired renal blood flow and clinically significant cytotoxic effects.^6^ Signs and symptoms associated with NSAID use that can complicate blood pressure management, such as hypertension and edema, are relatively infrequent^5^ but important.

Most epidemiologic research on the association of NSAIDs and incident kidney disease has involved older persons and/or those with chronic and serious conditions.^7,8,9,10,11,12,13^ Particularly regarding chronic and end-stage kidney disease, NSAID-related research has often focused on specific areas, such as disease progression.^14,15^ For younger healthy individuals, some studies provide statements of reassurance about the overall risks of NSAIDs^16^ and, in particular, about their renal effects.^17^ However, evidence on this demographic group is relatively sparse. This limited information may be because NSAID use is less common among young and middle-aged adults,^1^ and the expected population rate of clinically significant kidney disease due to NSAIDs is less than 1%.^18^

Studying the NSAID-kidney disease association among working-aged adults therefore requires a large group with robust NSAID use. United States Army soldiers are a useful study population given recent research indicating that 69% or more of this sizable population may use NSAIDs.^19^ In addition, prior studies have raised concerns about kidney disease risk among NSAID users who engage in endurance exercise,^20,21,22^ as renal blood flow may fall to as little as 25% of resting values during strenuous activity.^23^ The Army population is one in which endurance activities, such as running^24^ and long-distance rucksack marching,^25^ are regularly undertaken, so this group provides a unique window on NSAIDs and kidney disease among active persons. Other advantages of using a military population include standardized, comprehensive administrative and medical data, as well as preservice, annual, and combat duty–associated health screenings^26^ that facilitate recognition of incident diseases.

We therefore used data on the total active-duty US Army to estimate the independent associations between prescribed oral NSAID use and incident acute kidney injury (AKI) and chronic kidney disease (CKD). Renal effects of NSAIDs have been shown to be dose dependent.^18^ Increased frequency and duration of NSAID use amplify the risk of nonrenal adverse effects.^18,27^ Accordingly, we devised methods to study NSAID exposure volume over time while controlling for major factors of potential relevance to kidney dysfunction.

## Methods

### Population and Data

This retrospective cohort study was conducted with longitudinal data on the active-duty US Army collected from January 1, 2011, to December 31, 2014. Data were combined from official sources (eTable 1 in the Supplement) and stripped of identifiers. Analyses were conducted from August 1 to November 30, 2018. The institutional review board of Stanford University approved this study, which underwent secondary review by the Human Research Protections Office of the Defense Health Agency. A waiver of consent was granted because the research (1) involves no more than minimal risk to the participants, (2) does not affect the rights or welfare of the participants, and (3) could not practically be carried out without the waiver of consent. The study followed the Strengthening the Reporting of Observational Studies in Epidemiology (STROBE) reporting guideline.^28^

To facilitate time-to-event analyses, we used a person-month–based data set in which each participant was censored from further observation either after the incident outcome or, if applicable, on discharge from military service. Owing to the health screening associated with initiating service,^26^ soldiers who began duty during 2011-2014 were considered at risk for outcomes for all of the observed time because known kidney conditions would usually disqualify an applicant.

However, for soldiers already on duty in 2011, observation for incident outcomes began at the earliest recorded health maintenance encounter in or after July 2011. We required at least 6 months of observable time prior to the month of such examinations to increase detection of prior kidney problems (a total of 7 months of observation). Only soldiers with no diagnoses of AKI prior to or at the health maintenance encounter were included in the AKI analytic population. Similarly, only soldiers with no indication of CKD prior to or at their health maintenance encounter were included in the CKD analytic population.

Of 827 265 active Army soldiers who served during 2011-2014, a total of 764 228 met eligibility criteria for at least 1 of the 2 end point–specific analyses. In the AKI analysis, there were 763 572 persons observed for 1 705 533 person-years (mean [SD], 2.1 [1.1] person-years; median, 2.4 person-years). The 763 654 participants included in the CKD analysis were observed for 1 705 944 person-years (mean [SD], 2.2 [1.1] person-years; median, 2.4 person-years). There were 763 178 participants present in both analyses.

### Dependent Variables

During 2011-2014, the Army used the *International Classification of Disease, Ninth Revision, Clinical Modification* (*ICD-9-CM*) system. We identified outcomes from diagnoses in outpatient and inpatient care by using *ICD-9-CM* codes for AKI (584.x, 586, 580.9) and CKD (581.x, 583.x, 585.x, 587), following the convention of prior studies.^6,9,29,30,31^ A dedicated data system (eProfile) is an additional repository outside the health record per se in which soldiers' duty-limiting health conditions are tracked.^32^ We therefore also defined outcomes by using eProfile entries noting relevant kidney conditions.

### Independent Variables

#### Demographic Factors

Multiple demographic factors were included to control for potential confounding. Sex and Hispanic ethnicity were binary variables. Running age in years and self-reported race were categorical covariates.

#### Administrative Factors

We controlled for socioeconomic status by using each participant's running military pay grade.^33^ Total service time was updated each person-month. Combat duty was included as a covariate because of the associated potential for an increase in outcome risk due to injury or surgery.

#### Biomedical Factors

We used an NSAID exposure variable based on dispensed prescription medications and agent-specific World Health Organization–defined daily doses (DDDs),^34^ which represent estimates of typical maintenance doses for adults.^35^ The categorical variable represented the mean of the total monthly NSAID DDDs dispensed in the 6 months preceding each observation. This rolling window was used to capture exposures that were sufficiently long but also recent with regard to expected kidney effects; the present month was excluded to reduce the potential for overdose as a causal mechanism.

The *ICD-9-CM* codes were used to identify histories of the potentially contributory conditions hypertension^36^ (401.x, 402.x, 405) and type 1 or 2 diabetes^37^ (250.x). We included a covariate for a remote history of rhabdomyolysis (728.88, 791.3; ≥6 months in the past), as its immediate association with kidney injury appears to be well established.^38^ We further controlled for body mass index^39^ by using its standard categories,^40^ plus a category for missing data. Other potentially contributory conditions (eg, systemic lupus erythematosus^41^) were explored, but were deemed too infrequent in this population for inclusion.

### Statistical Analysis

To characterize the types and quantities of NSAID exposures, we tabulated counts of specific agent classes dispensed to study participants and percentages thereof. Preregression analyses included χ^2^ tests of distribution differences for selected covariates. To estimate the independent associations of NSAIDs with the kidney outcomes, we used dedicated Cox proportional hazards regression models for AKI and CKD. We also computed the adjusted risk of each outcome for participants in each of the NSAID exposure categories. These figures were calculated by totaling the products of the Cox regression coefficients and the covariate values, which permitted a computation of the absolute risk differences among the NSAID exposure groups. We additionally performed Wald tests for the interaction between selected medical conditions and NSAID use. In all analyses, 2-sided α < .05 defined statistical significance. All analyses were conducted using Stata statistical software, version 14.2 (StataCorp).

## Results

Of the 764 228 total participants, 655 392 (85.8%) were men; mean (SD) age was 28.6 (7.9) years (median, 27.0 years; interquartile range [IQR], 22.0-33.0 years); and 238 168 (31.2%) were new to the military during 2011-2014. There were 1 630 694 distinct NSAID prescriptions dispensed to participants during the total observation period, or a mean (SD) 2.1 (2.7) total prescriptions per person (median, 1). A total of 502 527 participants (65.8%) were not dispensed prescription NSAIDs in the prior 6 months, 137 108 (17.9%) were dispensed 1 to 7 mean total DDDs per month, and 124 594 (16.3%) received more than 7 DDDs per month. The mean (SD) DDD per prescription was 1.6 (1.0) (median, 2; IQR, 1.0-2.0). Ibuprofen and naproxen were the most commonly prescribed preparations and together accounted for 1 180 549 (72.4%) of the NSAIDs dispensed (Table 1). Of the 804 471 ibuprofen prescriptions, 78.3% were for 800-mg tablets, and 88.4% allowed for 3 or more daily doses. Of the 376 078 naproxen prescriptions, 95.7% were for 500-mg or stronger tablets, and 93.8% allowed for at least twice-daily use.

**Table 1.** Top NSAIDs Dispensed to 764 228 Study Participants

Agent^a^	NSAID Class	Prescriptions, No. (%)^b^
Ibuprofen	Propionic acid derivative	804 471 (49.3)
Naproxen	Propionic acid derivative	376 078 (23.1)
Meloxicam	Enolic acid derivative	176 638 (10.8)
Celecoxib	Selective COX-2 inhibitor	119 680 (7.3)
Acetylsalicylic acid	Salicylate (aspirin)	35 949 (2.2)
Diclofenac	Acetic acid derivative	34 118 (2.1)
Ketorolac	Acetic acid derivative	27 236 (1.7)
Indomethacin	Acetic acid derivative	24 795 (1.5)
Piroxicam	Enolic acid derivative	18 237 (1.1)
Etodolac	Acetic acid derivative	7142 (0.4)
Other	Multiple	6350 (0.4)

Abbreviations: COX-2, cyclooxygenase 2; NSAIDs, nonsteroidal anti-inflammatory drugs.

^a^ Suffix elements and compounds associated with the active component of applicable agents (eg, sodium) were omitted for simplicity.

^b^ There were 1 630 694 prescriptions dispensed in total during the observed time. Percentages may not total 100 owing to rounding.

There were 763 752 participants eligible for the AKI analysis, among whom 2356 (0.3%) experienced incident AKI events. Among the AKI outcomes, 13 (0.6%) were detected from eProfile data rather than diagnoses in the electronic health record. Of 763 654 individuals eligible for the CKD analysis, 1634 (0.2%) experienced incident CKD, including 9 cases (0.6%) solely detected via eProfile.

Histories of diabetes or rhabdomyolysis were present among fewer than 1% of the participants, while hypertension was more prevalent at up to 8.8%. We did, however, observe statistically significant differences in the distributions of biomedical and demographic factors comparing groups with and without NSAID exposure (Table 2). The proportion of women increased from 12.5% of those without NSAID use to 18.3% of those in the highest use group. Individuals who received the greatest NSAID volumes were twice as likely to be obese, composing 23.6% and 12.4% of the highest and lowest NSAID categories, respectively. Individuals who received the greatest NSAID volumes were also twice as likely to have histories of hypertension (8.8% vs 3.6% of the highest and lowest NSAID categories) and diabetes (0.9% vs 0.3% of the highest and lowest NSAID categories). African American participants were more highly represented among those who received the highest level of prescription NSAIDs than those who received none (22.9% vs 19.6%) (Table 2). Statistically significant differences in distributions were also observed for each of the military-specific factors. For example, increasing duration of military service was associated with increased NSAID use. Specifically, those with greater than 12 years of service made up 19.4% of the no NSAIDs group and 30.4% of the highest NSAIDs group (eTable 2 in the Supplement).

**Table 2.** Description of the 764 228 Participants at the Final Observation^a^

Factor^b^	No. (%)			*P* Value for χ^2^ Test^c^
	No NSAID	NSAID DDDs		
		1-7	>7	
Total	502 527 (65.8)	137 107 (17.9)	124 594 (16.3)	
Sex				
Male	439 916 (87.5)	113 708 (82.9)	101 768 (81.7)	<.001
Female	62 611 (12.5)	23 399 (17.1)	22 826 (18.3)	
Race				
White	356 886 (71.0)	91 898 (67.0)	83 813 (67.3)	<.001
African American	98 650 (19.6)	31 848 (23.2)	28 520 (22.9)	
Asian/Pacific Islander	24 818 (4.9)	6635 (4.8)	5025 (4.0)	
Native American	3969 (0.8)	1102 (0.8)	1077 (0.9)	
Other or unknown	18 204 (3.6)	5624 (4.1)	6159 (4.9)	
Hispanic ethnicity				
No	441 914 (87.9)	120 208 (87.7)	110 027 (88.3)	<.001
Yes	60 613 (12.1)	16 899 (12.3)	14 567 (11.7)	
Age, y				
≤22	177 029 (35.2)	41 811 (30.5)	29 278 (23.5)	<.001
23-27	135 509 (27.0)	37 912 (27.7)	30 355 (24.4)	
28-35	104 630 (20.8)	29 672 (21.6)	28 020 (22.5)	
36-41	49 478 (9.8)	15 206 (11.1)	18 488 (14.8)	
42-49	30 793 (6.1)	10 335 (7.5)	15 241 (12.2)	
≥50	5088 (1.0)	2171 (1.6)	3212 (2.6)	
Experienced acute kidney injury				
No	501 176 (99.7)	136 590 (99.6)	124 106 (99.6)	<.001
Yes	1351 (0.3)	517 (0.4)	488 (0.4)	
Experienced chronic kidney disease				
No	501 664 (99.8)	136 737 (99.7)	124 193 (99.7)	<.001
Yes	863 (0.2)	370 (0.3)	401 (0.3)	
BMI				
<18.5 (Underweight)	1623 (0.3)	529 (0.4)	452 (0.4)	<.001
18.5-24.99 (Normal)	155 863 (31.0)	44 472 (32.4)	33 110 (26.6)	
25.0-29.99 (Overweight)	205 591 (40.9)	62 586 (45.7)	58 806 (47.2)	
≥30.0 (Obese)	62 075 (12.4)	24 305 (17.7)	29 361 (23.6)	
Unknown	77 375 (15.4)	5215 (3.8)	2865 (2.3)	
Any observed history of hypertension				
No	484 500 (96.4)	128 523 (93.7)	113 605 (91.2)	<.001
Yes	18 027 (3.6)	8584 (6.3)	10 989 (8.8)	
Any observed history of diabetes				
No	500 814 (99.7)	135 976 (99.2)	123 516 (99.1)	<.001
Yes	1713 (0.3)	1131 (0.8)	1078 (0.9)	
History of rhabdomyolysis ≥6 mo prior				
No	501 714 (99.8)	136 854 (99.8)	124 340 (99.8)	.003
Yes	813 (0.2)	253 (0.2)	254 (0.2)	

Abbreviations: BMI, body mass index (calculated as weight in kilograms divided by height in meters squared); DDDs, defined daily doses; NSAID, nonsteroidal anti-inflammatory drug.

^a^ Column percentage totals may not total 100 owing to rounding.

^b^ eTable 2 in the Supplement provides other descriptive data on military service time, pay grade, and combat experience.

^c^ The *P* values indicate results of χ^2^ tests comparing factor distributions for those that were and were not found in each NSAID exposure category.

Results of analyses addressing the primary study aim are reported in Table 3. NSAID exposure of 7 or more DDDs per month was associated with significant increases in the adjusted hazard ratios (aHRs) of both AKI (aHR, 1.2; 95% CI, 1.1-1.4) and CKD (aHR, 1.2; 95% CI, 1.0-1.3). Based on postregression-adjusted risk computations, the highest NSAID exposure level was associated with annual case excesses per 100 000 exposed individuals of 17.6 cases for AKI and 30.0 cases for CKD. Mean NSAID exposure of 1 to 7 DDDs was associated with smaller hazard increases that were not significant.

**Table 3.** Analysis of Associations Between NSAID Use and Kidney Disease^a^

Factor	aHR (95% CI)	
	Acute Kidney Injury	Chronic Kidney Disease
Total DDDs prescribed per month in the prior 6 mo, mean		
0	1 [Reference]	1 [Reference]
1-7	1.1 (1.0-1.2)	1.1 (0.9-1.3)
>7	1.2 (1.1-1.4)^b^	1.2 (1.0-1.3)^c^
BMI		
<18.5 (Underweight)	1.1 (0.5-2.7)	2.0 (0.7-5.3)
18.5-24.99 (Normal)	1 [Reference]	1 [Reference]
25.0 to 29.99 (Overweight)	1.2 (1.1-1.4)	1.1 (1.0-1.3)^b^
≥30.0 (Obese)	1.5 (1.3-1.7)^b^	1.6 (1.3-1.8)^b^
Unknown	0.7 (0.5-0.8)^b^	0.4 (0.3-0.6)^b^
History of hypertension		
Yes	3.2 (2.9-3.6)^b^	4.5 (4.0-5.1)^b^
No	1 [Reference]	1 [Reference]
History of diabetes		
Yes	1.8 (1.4-2.4)^b^	1.8 (1.4-2.2)^b^
No	1 [Reference]	1 [Reference]
History of rhabdomyolysis >6 mo		
Yes	2.9 (1.9-4.7)^b^	2.7 (1.7-4.4)^b^
No	1 [Reference]	1 [Reference]
Sex		
Male	2.3 (2.0-2.7)^b^	1.6 (1.4-1.9)^b^
Female	1 [Reference]	1 [Reference]
Race		
White	1 [Reference]	1 [Reference]
African American	1.6 (1.4-1.7)^b^	2.3 (2.0-2.5)^b^
Asian/Pacific Islander	0.9 (0.8-1.2)	1.1 (0.9-1.4)
Native American	0.9 (0.6-1.5)	0.3 (0.1-1.0)
Other or unknown	1.1 (0.9-1.4)	1.1 (0.8-1.4)
Hispanic ethnicity		
Yes	0.8 (0.6-0.9)^d^	1.0 (0.8-1.2)
No	1 [Reference]	1 [Reference]
Age, y		
≤22	1 [Reference]	1 [Reference]
23-27	1.3 (1.1-1.5)^d^	1.5 (1.1-2.0)^d^
28-35	1.5 (1.2-1.7)^b^	2.1 (1.6-3.0)^b^
36-41	1.8 (1.5-2.2)^b^	3.7 (2.7-5.2)^b^
42-49	2.1 (1.7-2.6)^b^	5.0 (3.5-7.1)^b^
≥50	3.1 (2.3-4.1)^b^	7.1 (4.8-10.4)^b^

Abbreviations: aHR, adjusted hazard ratio; BMI, body mass index (calculated as weight in kilograms divided by height in meters squared); DDDs, defined daily doses; NSAID, nonsteroidal anti-inflammatory drug.

^a^ Cox proportional hazards regression models used in analyses. The models additionally controlled for military service time, pay grade, and combat experience. eTable 3 in the Supplement provides related findings.

^b^ *P* < .001.

^c^ *P* < .05.

^d^ *P* < .01.

Obesity was associated with significant increases in the hazard of each outcome (AKI: aHR, 1.5; 95% CI, 1.3-1.7; CKD: aHR, 1.6; 95% CI, 1.3-1.8), and overweight status was also associated with a modest, significant increase in the hazard of AKI (aHR, 1.2; 95% CI, 1.1-1.4). Histories of hypertension (AKI: aHR, 3.2; 95% CI, 2.9-3.6; CKD: HR, 4.5; 95% CI, 4.0-5.1) and rhabdomyolysis (AKI: aHR, 2.9; 95% CI, 1.9-4.7; CKD: aHR, 2.7; 95% CI, 1.7-4.4) were each associated with greater than 2-fold increases in the adjusted hazard of both outcomes, while diabetes conferred smaller increases (AKI: aHR, 1.8; 95% CI, 1.4-2.4; CKD: aHR, 1.8; 95% CI, 1.4-2.2) (Table 3). Statistically significant associations with kidney outcomes were also observed for multiple demographic factors. Male sex was associated with more than twice the adjusted hazard of AKI (aHR, 2.3; 95% CI, 2.0-2.7) and a smaller but significant increase in the CKD hazard (aHR, 1.6; 95% CI, 1.4-1.9). African American participants had more than twice the hazard of CKD (aHR, 2.3; 95% CI, 2.0-2.5) compared with white participants, and a smaller, significant increase for AKI (aHR, 1.6; 95% CI, 1.4-1.7). Participants of Hispanic ethnicity had a lower adjusted hazard of AKI (aHR, 0.8; 95% CI, 0.6-0.9) relative to other ethnicities.

Participants older than 22 years had a higher adjusted hazard of each outcome compared with younger participants. The association with age was strongest in the CKD analysis, where those aged 42 to 49 years experienced a 5.0-fold hazard increase (95% CI, 3.5-7.1), and individuals 50 years or older experienced a 7.1-fold increase (95% CI, 4.8-10.4). Statistically significant hazard increases were also found in association with some military-specific factors (eTable 3 in the Supplement).

To address the issue of whether the selected medical condition covariates might interact with NSAID use, we conducted a formal test of the statistical significance of each such interaction (hypertension, diabetes, and rhabdomyolysis). Only the interaction between prior hypertension and NSAIDs in the CKD analysis was statistically significant (aHR, 0.7; 95% CI, 0.5-0.9). This finding provides some evidence that, in this population, the association between NSAIDs and CKD is significantly weaker among those with prior hypertension than those without.

## Discussion

In this study we identified modest but statistically significant associations between the highest level of dispensed NSAIDs and incident AKI and CKD in a large military population. Specifically, the adjusted hazard of each outcome was approximately 20% higher among participants who received more than 7 total NSAID DDDs per month compared with those who did not receive prescription NSAIDs. This level of use was associated with 17.6 and 30.0 additional cases per exposed 100 000 persons per year for AKI and CKD, respectively. These potentially preventable cases are of particular concern in a population in which medical readiness is a foundation of national security. Because most participants were younger than 35 years and free of hypertension, diabetes, and/or rhabdomyolysis, this study provided an unusual opportunity to evaluate young, healthy, active adults who received relatively high NSAID doses (mean, 1.6 DDDs per prescription). No significant elevation in risk was observed among soldiers prescribed between 1 and 7 DDDs of NSAIDs per month.

The NSAID-related risk estimates for AKI in other studies have ranged from approximately 2- to 8-fold increases,^8,9,10^ which are higher than what we found in our analysis. Our risk estimates for CKD associated with NSAIDs were relatively similar to those seen in one analysis,^12^ but lower than the doubling of risk reported elsewhere.^7^ Direct comparison with past studies is challenging because most have focused on older patients and those with comorbidities, and also because of varying outcome and exposure definitions.

Other findings included elevated hazards of incident AKI and CKD with increasing age and among men and African American participants. Our CKD findings differed from those seen in the United States Renal Data System, where women demonstrated a higher CKD rate.^42^ Hispanic soldiers had a lower hazard of AKI compared with non-Hispanic individuals.

### Strengths and Limitations

Strengths of this study include the use of standardized, detailed data on a large population and the ability to exclude those with prior disease. Our results may generalize reasonably well to nonmilitary adults of similar ages, but exposures among service members might differ substantially from those of civilians. In addition to required physical exertion, the life of most Army soldiers includes regular field training in outdoor settings. Most US Army installations are in the warm US south,^43^ and recent combat deployments have taken place in largely hot and arid regions. Therefore, intermittent dehydration that further depletes fluid volume and increases the strain on the kidneys^44^ may be unusually frequent or substantial among soldiers. Our study's results may most closely apply to civilians with strenuous, potentially dehydration-producing occupations, such as athletes, firefighters, and farm, construction, and industrial workers.

This research was subject to the limitations of diagnosis coding, including general imprecision. One concern associated with diagnosis code validity could be case underdetection,^45,46^ which may arise when procedure codes, such as for kidney transplantation, are entered rather than kidney disease codes.^47^ This issue is likely less important in our study population because early and accurate identification of serious conditions is a key duty of military clinicians to ensure adherence to medical service standards for training and duty.^26^ A diagnosis would usually occur well before advanced procedures, such as hemodialysis or transplantation, are required. Also, our data sets afforded somewhat augmented event detection owing to clinician entries in the eProfile record system.

We nonetheless acknowledge that our CKD case detection mechanisms may have been reduced by our relatively short follow-up times, as clinical diagnoses and eProfile entries may have occurred afterward for some participants. Misclassification of AKI as CKD and vice versa constitutes another specific possible form of potential imprecision in our data, but the similar findings for the outcomes reduce this concern. We also acknowledge that the sensitivity and specificity of diagnosis codes may further vary in unknown ways, such as across exposure strata. More generally, as in any observational study, residual confounding is possible. However, the wide array of demographic, job-related, and health-related control variables used should reduce concerns.

Other limitations of the study arise from our reliance on dispensed NSAID prescriptions to quantify drug exposure. Whereas our data captured clinicians' instructions, there was no mechanism to observe the details of individual NSAID use. We would expect this approach to have created conservative association estimates because if prescription NSAID intake varied from the total quantity prescribed, it was presumably lower. However, we were unable to account for over-the-counter NSAID use, which could have offset this phenomenon.

Of the participants, 238 168 (31.2%) were new to the Army during the observation period. These individuals differed in gross exposure to the military environment from those with greater total service times. Furthermore, the presence of experienced soldiers in the data set represents a possible selection sieve, as these soldiers have served for potentially many years. We included the covariates for age, service time, and combat experience specifically to provide control for these factors.

Recently, a more cautious tone has permeated the discussion about NSAID use,^45,48,49^ with concerns including the potential delayed or inhibited healing associated with pain management.^50^ Nonpharmacologic interventions are increasingly emphasized,^51^ and research evidence on such options is available.^52^ Our findings provide additional support for the need for expanded research on alternative treatment options for pain and a greater focus on patient education about the risks and benefits of higher doses of NSAIDs.

## Conclusions

We have identified modest but statistically significant associations between the highest level of observed NSAID exposure and incident AKI and CKD among active, largely healthy adults in the military. While recognizing that the pain burden in such active populations must be managed using the best-available measures, given the relatively high mean DDD per prescription we observed, providing lower doses is one approach to those with pain and/or inflammation. The increases in kidney disease risk that we observed for modifiable factors, such as body mass index and hypertension, reinforce the established importance of managing these conditions, regardless of patient age.

## Notes

Supplement. **eTable 1.** Descriptions of the Data Sources Leveraged to Produce the Research Datasets **eTable 2.** Raw Counts (Percentages) of Subjects (N = 764,228) Organized by Traits, Outcomes and Drug Exposure at the Last Observation **eTable 3.** Adjusted Hazard Ratios (HRs) and Statistical Significance Indicators From Cox Proportional Hazards Regression Models
