# Supplementary material for: Association of Nonsteroidal Anti-inflammatory Drug Prescriptions With Kidney Disease Among Active Young and Middle-aged Adults
Source: JAMA Netw Open. 2019 Feb 15;2(2):e187896. doi: 10.1001/jamanetworkopen.2018.7896 (PMC6484592; doi:10.1001/jamanetworkopen.2018.7896)
Supplement: Supplement. — eTable 1. Descriptions of the Data Sources Leveraged to Produce the Research Datasets eTable 2. Raw Counts (Percentages) of Subjects (N = 764,228) Organized by Traits, Outcomes and Drug Exposure at the Last Observation eTable 3. Adjusted Hazard Ratios (HRs) and Statistical Significance Indicators From Cox Proportional Hazards Regression Models [file jamanetwopen-2-e187896-s001.pdf]

Supplementary Online Content

Nelson DA, Marks ES, Deuster PA, O'Connor FG, Kurina LM. Association of nonsteroidal anti-inflammatory drug prescriptions with kidney disease among active young and middle-aged adults. *JAMA Netw Open*. 2019;2(2):e187896. doi:10.1001/jamanetworkopen.2018.7896

eTable 1. Descriptions of the Data Sources Leveraged to Produce the Research Datasets

eTable 2. Raw Counts (Percentages) of Subjects (N = 764,228) Organized by Traits, Outcomes and Drug Exposure at the Last Observation

eTable 3. Adjusted Hazard Ratios (HRs) and Statistical Significance Indicators From Cox Proportional Hazards Regression Models

This supplementary material has been provided by the authors to give readers additional information about their work.

© 2019 Nelson DA et al. *JAMA Network Open.*

eTable 1. Descriptions of the Data Sources Leveraged to Produce the Research Datasets.

Defense Manpower Data Center (DMDC)[1]
• Active Duty Master File – From official Department of Defense (DoD) records of demographic and military service data, including pay grades<br>• Transactions File – From official DoD records of military duty status changes, such as completion of service that may define the end of observation
Military Health System Data Repository (MDR)[2]
• Combined Ambulatory Professional Encounter Record: From digital records on outpatient care in military facilities<br>• Tricare Encounter Data, Non-Institutional: From digital records on outpatient care in civilian facilities that was reimbursed by military health coverage<br>• Standard Inpatient Data Record: From digital records on inpatient care in military facilities<br>• Tricare Encounter Data, Institutional: From digital records on inpatient care in civilian facilities that was reimbursed by military health coverage<br>• Clinical Data Repository Vitals File: From digital records of height and weight readings at outpatient encounters in military facilities<br>• Pharmacy Detail Transaction Service: From digital records on medications dispensed by military or civilian pharmacies

© 2019 Nelson DA et al. *JAMA Network Open.*

Medical Operational Data System (MODS)[3]
• Periodic Health Assessment: Self-report and clinician-entered data from required annual health screenings at military health facilities<br>• eProfile: Data from the official electronic archive of formal duty and activity restrictions or "profiles," with associated clinical reasons
Digital Training Management System (DTMS)[4]
• Army Body Composition Program: Readings from required, biannual height and weight assessments

1. Department of Defense (DoD) Web site. *DMDC* (Defense Manpower Data Center) [Internet]. Washington (DC): DoD; 2018 [cited 2018 January 22]. https://www.dmdc.osd.mil/appj/dwp/index.jsp. Accessed November 14, 2018.

2. Military Health System Web site. *Military Health System Data Repository*. [Internet]. Washington (DC): Department of Defense; 2018 [cited 2018 January 22]. https://health.mil/Military-Health-Topics/Technology/Clinical-Support/Military-Health-System-Data-Repository. Accessed November 14, 2018.

3. US Army Medical Department. *Consultant Toolkit: Medical Operational Data System (MODS)*. http://medicalservicecorps.amedd.army.mil/toolkit.html. Accessed November 14, 2018.

4. US Army Combined Arms Center. *Digital Training Management System.* August 18, 2017. https://usacac.army.mil/organizations/cact/news/01-20-2015_digital-training-management-system. Accessed November 14, 2018.

© 2019 Nelson DA et al. *JAMA Network Open.*

eTable 2. Raw Counts (Percentages[A]) of Subjects (N = 764,228) Organized by Traits, Outcomes and Drug Exposure at the Last Observation.

**Factors[B]**	**No NSAIDs 502,527 (65.8)**	**One to seven NSAID DDDs 137,108 (17.9)**	**Greater than seven NSAID DDDs 124,594 (16.3)**	***P*-value[C] for chi square test**
Military pay grade				
Privates (E-1 to E-3)	167,309 (33.3)	37,652 (27.5)	27,106 (21.8)	< 0.001
Specialists & corporals (E-4)	107,982 (21.5)	38,648 (28.2)	34,778 (27.9)	
Junior sergeants (E-5 to E-6)	109,046 (21.7)	33,476 (24.4)	31,161 (25.0)	
Senior sergeants (E-7 to E-9)	39,015 (7.8)	11,574 (8.4)	15,356 (12.3)	
Warrant officers (W-1 to W-5)	11,467 (2.3)	2837 (2.1)	3254 (2.6)	
Junior officers (O-1 to O-3)	44,164 (8.8)	7504 (5.5)	5855 (4.7)	
Senior officers (≥O-4)	23,544 (4.7)	5416 (4.0)	7084 (5.7)	
Military service time, years				
≤3	220,934 (44.0)	50,804 (37.1)	36,249 (29.1)	< 0.001
>3 to 6	92,389 (18.4)	30,140 (22.0)	26,138 (21.0)	
>6 to 12	91,746 (18.3)	26,239 (19.1)	24,368 (19.6)	
>12	97,458 (19.4)	29,924 (21.8)	37,839 (30.4)	

© 2019 Nelson DA et al. *JAMA Network Open.*

Number of combat deployments				
None	216,725 (43.1)	53,031 (38.7)	42,474 (34.1)	< 0.001
1	123,893 (24.7)	38,741 (28.3)	35,062 (28.1)	
2	78,658 (15.7)	23,303 (17.0)	23,465 (19.0)	
≥3	83,251 (16.6)	22,032 (16.1)	23,413 (18.8)	

A. Column percentage totals may not equal 100% due to rounding.
B. See Table 2 for descriptive data on gender, race, Hispanic ethnicity, Body Mass Index and histories of hypertension, diabetes mellitus and rhabdomyolysis.
C. The *P*-values indicate results of chi square tests comparing factor distributions across the NSAID use categories.

© 2019 Nelson DA et al. *JAMA Network Open.*

eTable 3. Adjusted Hazard Ratios (HRs) and Statistical Significance Indicators From Cox Proportional Hazards Regression Models.[A]

Factors	Acute kidney injury	Chronic kidney disease
	HR (95% CI)	HR (95% CI)
Military pay grade		
Privates (E-1 to E-3)	1.9*** (1.4 – 2.6)	0.8 (0.6 – 1.2)
Specialists & corporals (E-4)	1.5*** (1.2 – 1.9)	1.0 (0.8 – 1.3)
Junior sergeants (E-5 to E-6)	1.5*** (1.2 – 1.8)	0.9 (0.7 – 1.1)
Senior sergeants (E-7 to E-9)	1.2* (1.0 – 1.5)	1.0 (0.9 – 1.2)
Warrant officers (W-1 to W-5)	0.9 (0.7 – 1.2)	0.8 (0.7 – 1.1)
Junior commissioned officers (O-1 to O-3)	1.5** (1.2 – 1.9)	0.8 (0.6 – 1.0)
Senior commissioned officers (≥O-4; reference)	1.0	1.0
Military service time, years		
≤3 (reference)	1.0	1.0
>3 to 6	1.2 (1.0 – 1.4)	1.3 (1.0 – 1.8)
>6 to 12	1.2 (0.9 – 1.5)	1.7** (1.2 – 2.3)
>12	1.1 (0.90 – 1.4)	1.8** (1.3 – 2.6)

© 2019 Nelson DA et al. *JAMA Network Open.*

Number of combat deployments (referent: ≥3)		
0	1.3** (1.1 – 1.5)	1.4*** (1.2 – 1.6)
1	1.0 (0.9 – 1.2)	1.2* (1.0 – 1.3)
2	1.0 (0.9 – 1.1)	1.1 (1.0 – 1.3)

A. The models additionally controlled for non-steroidal anti-inflammatory medication exposure, Body Mass Index, prior histories of hypertension, diabetes and rhabdomyolyis, gender, race, Hispanic ethnicity, and age. See Table 3 for the related findings.

© 2019 Nelson DA et al. *JAMA Network Open.*
